# Delivery of cancer therapies by synthetic and bio-inspired nanovectors

**DOI:** 10.1186/s12943-021-01346-2

**Published:** 2021-03-24

**Authors:** Tina Briolay, Tacien Petithomme, Morgane Fouet, Nelly Nguyen-Pham, Christophe Blanquart, Nicolas Boisgerault

**Affiliations:** Université de Nantes, Inserm, CRCINA, F-44000 Nantes, France

**Keywords:** Cancer therapy, Vectorization, Nanomedicine, Drug delivery, Targeting, Virus, Nanoparticle, Vesicle

## Abstract

**Background:**

As a complement to the clinical development of new anticancer molecules, innovations in therapeutic vectorization aim at solving issues related to tumor specificity and associated toxicities. Nanomedicine is a rapidly evolving field that offers various solutions to increase clinical efficacy and safety.

**Main:**

Here are presented the recent advances for different types of nanovectors of chemical and biological nature, to identify the best suited for translational research projects. These nanovectors include different types of chemically engineered nanoparticles that now come in many different flavors of ‘smart’ drug delivery systems. Alternatives with enhanced biocompatibility and a better adaptability to new types of therapeutic molecules are the cell-derived extracellular vesicles and micro-organism-derived oncolytic viruses, virus-like particles and bacterial minicells. In the first part of the review, we describe their main physical, chemical and biological properties and their potential for personalized modifications. The second part focuses on presenting the recent literature on the use of the different families of nanovectors to deliver anticancer molecules for chemotherapy, radiotherapy, nucleic acid-based therapy, modulation of the tumor microenvironment and immunotherapy.

**Conclusion:**

This review will help the readers to better appreciate the complexity of available nanovectors and to identify the most fitting “type” for efficient and specific delivery of diverse anticancer therapies.

## Introduction

**Fig. 1:**
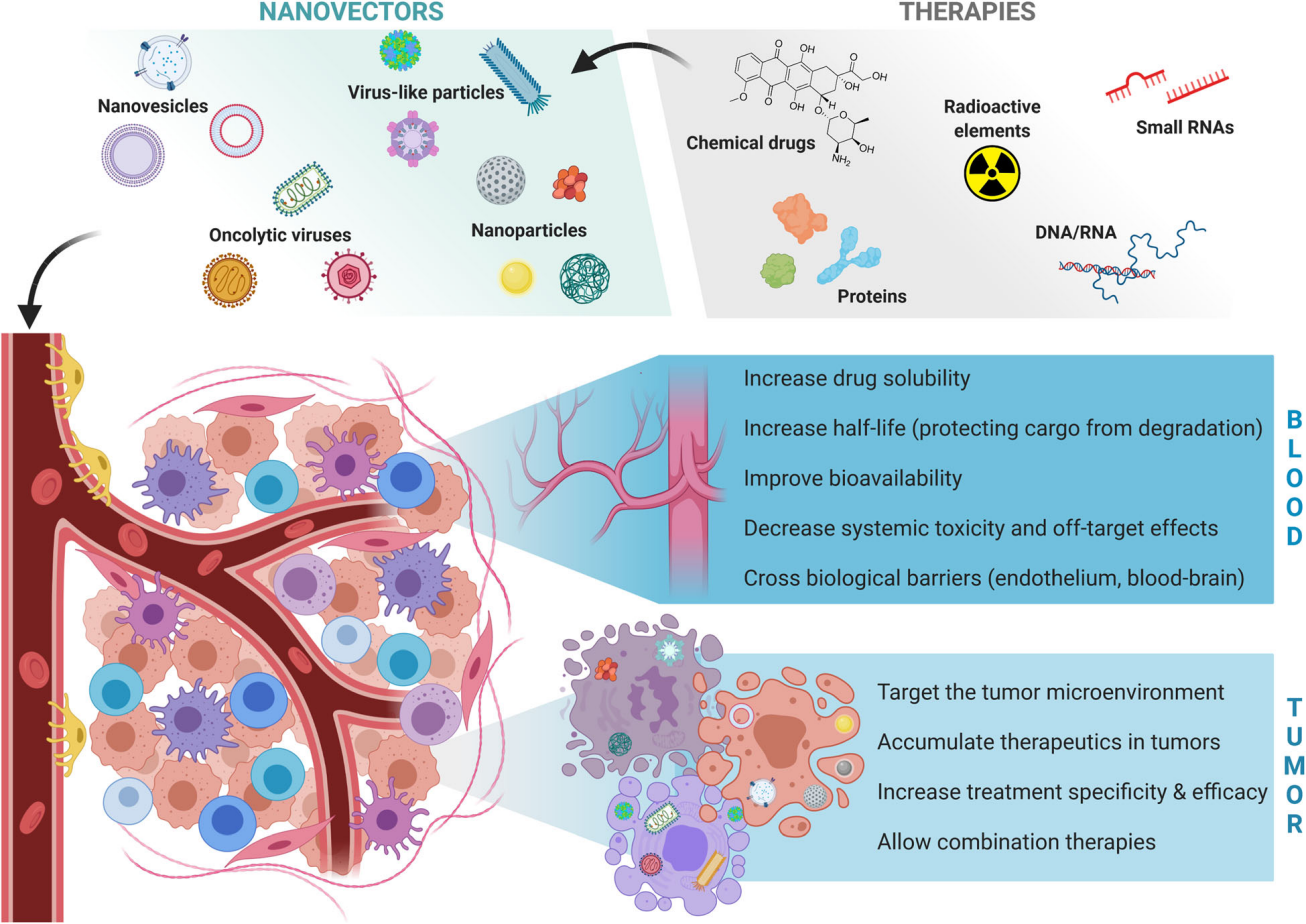
Advantages of vectorization for delivering cancer therapies. The clinical efficacy of therapeutic molecules (e.g. chemotherapeutic drugs, radionuclides, nucleic acids, antibodies) relies on efficient tumor delivery and limited off-targeting. Nanovectors of different natures (e.g. nanoparticles, extracellular vesicles, viruses) can improve the transport of these molecules in the bloodstream by increasing their solubility, half-life and bioavailability, and by helping the crossing of biological barriers. Tumor delivery is also enhanced by improved targeting of the tumor microenvironment, leading to the accumulation of the therapeutic molecules in the tumors and thus potentiating the use of combination therapies

Cancer causes approximately 10 million deaths per year worldwide for around 18 million new cases [1]. Advanced understanding of cancer biology and continuous improvement of treatments such as radiotherapy, chemotherapy and more recently immunotherapy have steadily ameliorated patient survival over the years. In many cases, these treatments remain associated with adverse effects and limited efficacy due to a lack of tumor specificity. Resistances to single treatments are commonly addressed by combination therapies that can further increase the risks of life-threatening toxicities. Moreover, some categories of molecules such as hydrophobic drugs, radioisotopes, toxins or nucleic acids cannot be injected systemically to patients because of their instability or of extensive off-target effects. These limitations can be overcome through vectorization using nanocarriers that will increase drug solubility and bioavailability, improve the targeting of the cancer microenvironment, augment local drug concentration in tumors and potentiate the efficacy of therapeutic combinations [2, 3] (Fig. 1).

**Fig. 2:**
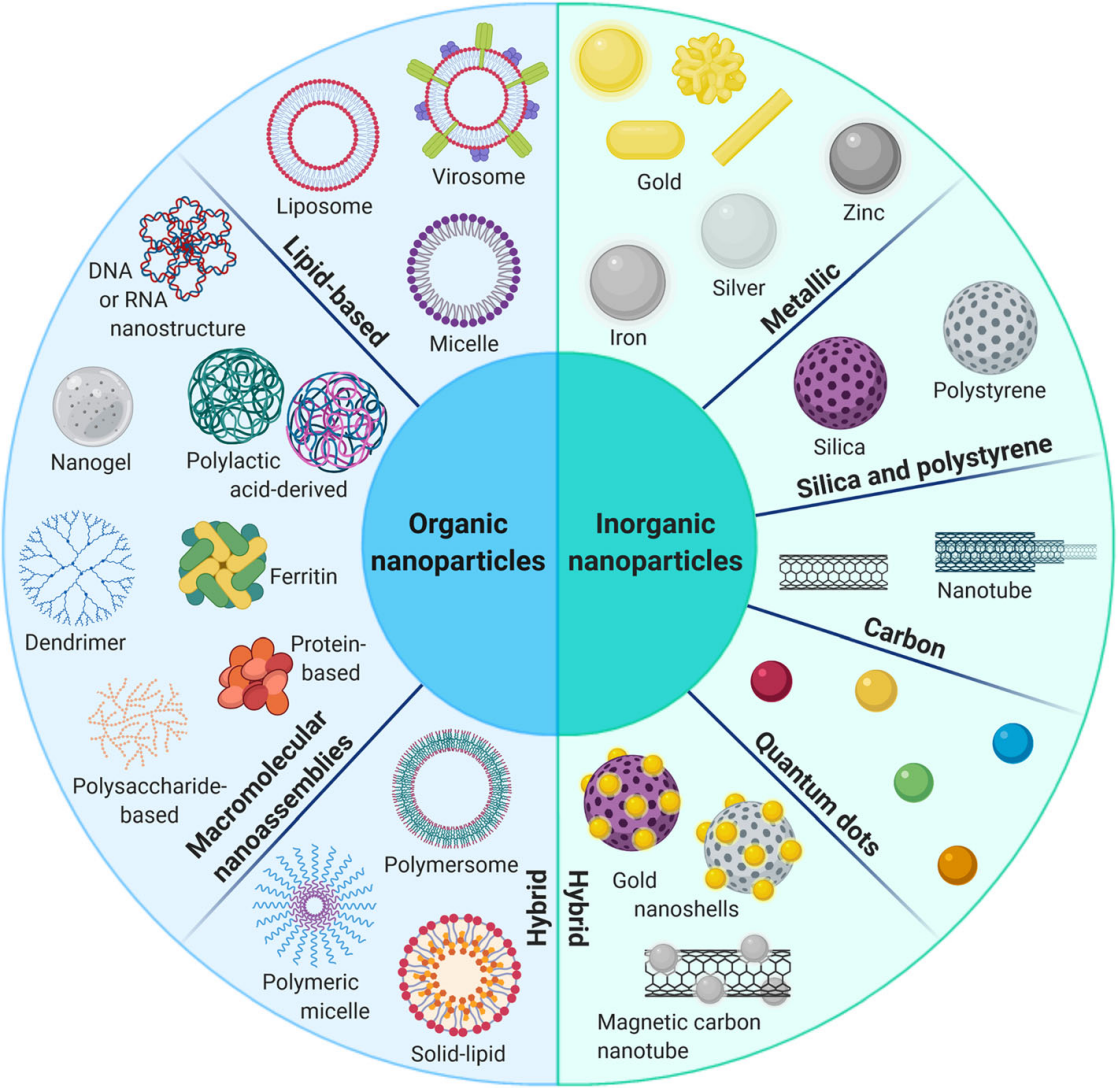
Chemically engineered nanoparticles for cancer therapy. This class of nanovectors is commonly divided between inorganic and organic nanoparticles. Inorganic nanoparticles (e.g. metallic, silica, carbon, quantum dots) are characterized by a high stability, a low biodegradability and intrinsic electronical and optical properties suitable for cancer imaging and theranostics. Because of their solid core, therapeutic molecules are generally conjugated on their surface and may be exposed to rapid degradation *in vivo*. Organic nanoparticles (e.g. lipid-based, macromolecular assemblies) exhibit a lower stability but a good biocompatibility and multiple possibilities of drug functionalization on their surface or their inner space. Hybrid nanoparticles combine the advantages of both inorganic and organic families to improve the biocompatibility and the stability of the nanovector

**Fig. 3:**
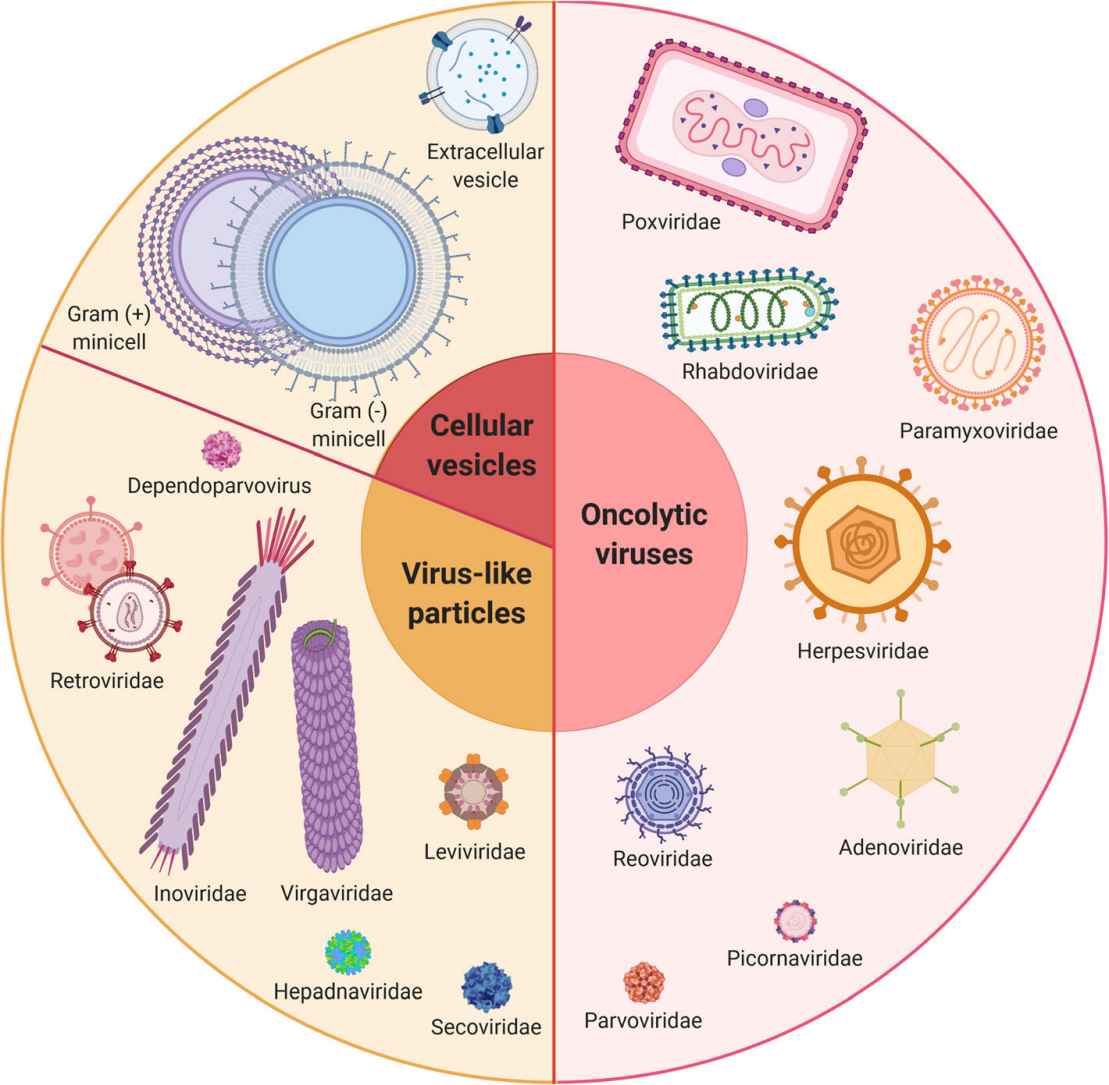
Biological and bio-inspired nanovectors for cancer therapy. These nanovectors have been derived from different types of organisms and exhibit high biocompatibility and extensive engineering possibilities. Extracellular vesicles derive from eukaryotic cell membranes and naturally transport different types of biomolecules (e.g. proteins, RNA). Bacterial minicells are achromosomal 400-nanometer vesicles that can be generated by genetic engineering of bacteria and have been recently used to vectorize various types of therapeutic molecules. Virus-like particles are basically viruses (e.g. bacteriophages, plant viruses, eukaryotic viruses) stripped of their replicative capacity; they exist as naked or enveloped capsids and sometimes require a non-replicative template genome for their assembly. On the contrary, oncolytic viruses are tumor-specific, live-replicating viruses with intrinsic cytotoxic and immunoactivating properties; they can equally be naked or enveloped and may be modified by genetic engineering to transport therapeutic transgenes that will be expressed exclusively by infected malignant cells

**Fig. 4:**
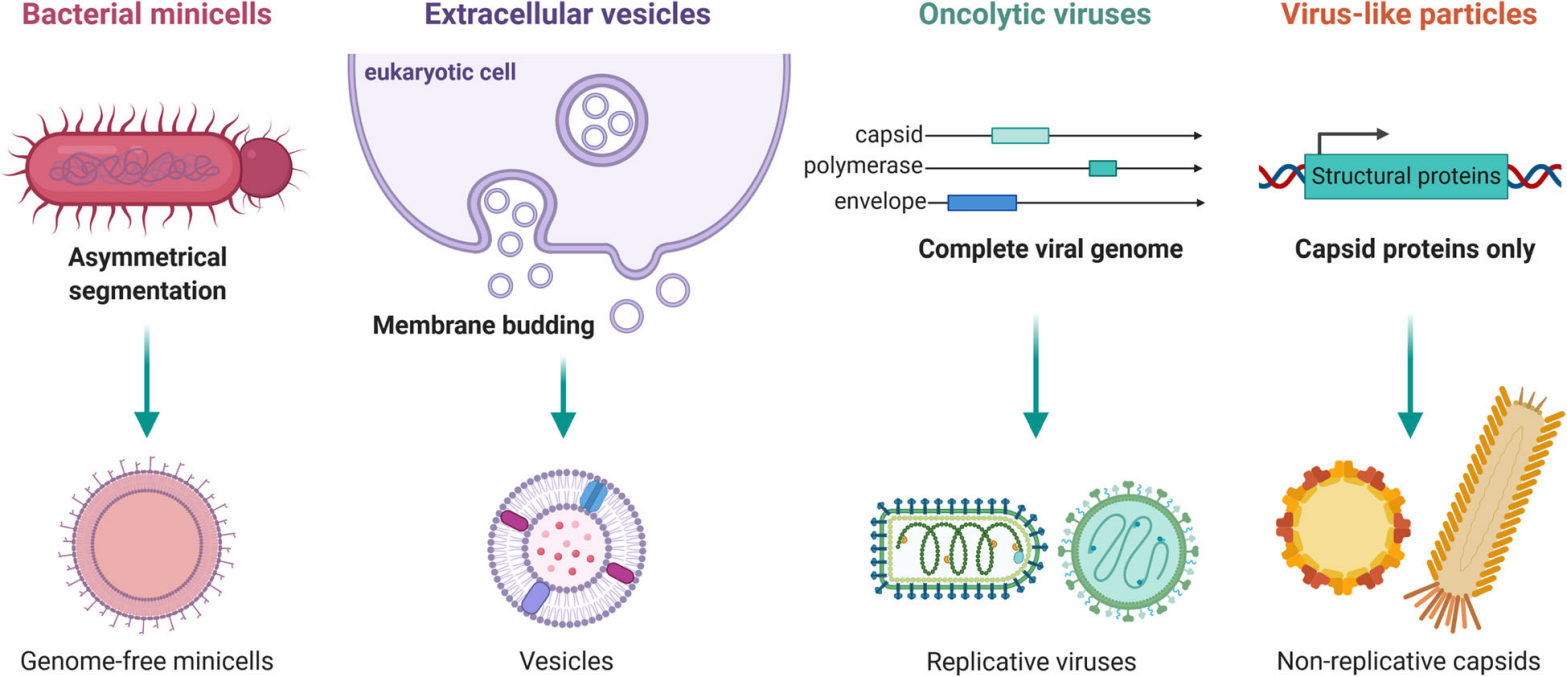
Biogenesis of biological nanovectors. Biological nanovectors are either derived from prokaryotic (bacterial minicells) or eukaryotic (extracellular vesicles) cells, or from viruses (oncolytic viruses and virus-like particles). Bacterial minicells are achromosomal vesicles obtained upon genetic engineering (deletion of the Min operon) from ectopic septation of Gram-positive or Gram-negative bacteria. Extracellular vesicles are produced by all eukaryotic cells by outward budding of the plasma membrane (microvesicles) or through inward budding and exocytosis (exosomes). Regarding viruses, whereas live-attenuated oncolytic viruses carry a complete genome and thus retain a replicative capacity specific for transformed cells, virus-like-particles are only constituted of structural proteins and are consequently not competent for replication

Specific targeting, which is key to increase treatment efficacy while reducing detrimental off-target effects, remains a major scientific challenge in multiple areas of therapeutic research. In cancer therapy, vectorization approaches have recently diversified with the development of new families of nanovectors (1 to 1,000 nm) created by chemical engineering (e.g. nanoparticles) [3] or derived from the biological world (e.g. bacteria, viruses, extracellular vesicles) [4]. Although this adds to the complexity of drug development, efficient vectorization appears as essential to further improve the safety and efficacy of both current and future cancer therapies. In this review, we chose to focus on nanovectors that are able to protect and to carry therapeutic payloads to tumors following a systemic injection. This does not include antibody-mediated vectorization [5], cancer vaccination strategies [6] or vectorization for imaging [7] – for instance for guided surgery – which have been reviewed elsewhere. We first introduce the various families of nanovectors available today, including the different subtypes of organic and inorganic nanoparticles (Fig. 2), cell-derived extracellular vesicles (EVs), virus-like particles (VLPs) (e.g. plant and animal viruses, bacteriophages), oncolytic viruses (OVs) and bacterial minicells (Figs. 3 and 4). These vectors display different physical and structural properties that dictate their abilities to be coupled to different types of therapeutic molecules (e.g. chemotherapeutic drugs, radioisotopes, proteins, nucleic acids) and make them adapted to different biological and clinical situations. A clear understanding of the advantages and limitations of each of these nanovectors (Table 1) to transport different therapeutic agents (Table 2) and of their evolving potential will help developing better vectorization approaches in the future.

**Table 1:** Main properties of the different families of nanovectors

**Nanovector family**	**Biocompatibility**	**Stealth**	**Immunogenicity**	**Ease of** **retargeting**	**Systemic injection**	**Frequent** **off-targets**	**Replicative**	**Stability**	**Standardized production**	**Cost**
***Inorganic nanoparticles***	Very low	Good	Low	High	Possible	Liver, spleen	No	Good	Adapted	$$$
***Organic nanoparticles***	Good	Good	Low	High	Adapted	Liver, spleen	No	Medium	Feasible	$$
***Extracellular vesicles***	High	High	None	Low	Adapted	Liver	No	Low	No	$$$
***Bacterial minicells***	High	Low	Medium	Medium	Adapted	Liver	No	Medium	Feasible	$
***Virus-like particles***	High	Medium	Medium	High	Adapted	Liver	No	Medium	Feasible	$$$
***Oncolytic viruses***	High	Medium	High	Low	Possible	Depends on virus tropism	Yes	Low	Difficult	$$$

**Table 2:** Suitability of the different families of nanovectors for the vectorization of anti-cancer therapeutics

**Nanovector family**	**Chemotherapy**	**Radiotherapy**	**Gene therapy**	**RNA interference**	**TME modification**	**Immunotherapy**
***Nanoparticles***	+++	++	+	+	+	+
***Extracellular vesicles***	+	-	+	++	NT	+
***Bacterial minicells***	++	NT	NT^*^	++	NT	NT
***Virus-like particles***	+	NT	+++	++	NT	+
***Oncolytic viruses***	-	+	+++	++	+++	+++

+++: optimal; ++: adapted; +: feasible; -: not adapted.

NT: never tested, TME: tumor microenvironment.

^*^ expected to be similar to RNA interference

## Types of nanovectors

### Nanoparticles

Chemically engineered nanoparticles form a vast class of nanovectors with a wide variety of structures, sizes and compositions [8, 9] (Fig. 2). Among the inorganic family, the most studied are metallic (e.g. gold, iron oxide) nanoparticles that display unique optical and electronic properties particularly favorable for biomedical imaging [10]. Because of their solid core, drug functionalization consists in surface bonding and exposes conjugated drugs to both degradation and exchange dynamics in the bloodstream. Their use in therapy is also limited by a low biodegradability. Mesoporous inorganic nanoparticles – mostly biodegradable, silica-based – constitute an alternative to protect drugs within a porous structure but their safety profile still needs characterization [11, 12]. On the other hand, the organic nanoparticle family exhibits better biocompatibility and biodegradability, making those more suitable for therapeutic applications. The first organic subfamily encompasses natural (e.g. protein- and polysaccharide-based) and synthetic (e.g. polylactic acid derivatives, dendrimers, fluorescent organic nanoparticles) macromolecular nanoassemblies (also improperly called polymeric nanoparticles) that possess a good stability and display numerous free functional groups endowing them with a high loading capacity [8, 13]. These properties explain the growing interest for such nanoassemblies in cancer therapy even if the *in vivo* characterization of each of their subunits remains challenging. The second organic subfamily contains lipid-based nanoparticles that are the most represented in preclinical and clinical studies due to their unmatched biocompatibility [8, 14, 15]. They basically consist in lipid monolayered (i.e. micelles) or bilayered (i.e. liposomes) nanovesicles and can vectorize a broad range of molecules with distinct physicochemical properties; hydrophobic drugs can be embedded within the lipid bilayer of liposomes or loaded in the core of micelles while hydrophilic drugs are either entrapped in the aqueous core of liposomes or displayed on their surface [16, 17]. However, lipid-based nanoparticles still face several limitations among which a low loading capacity and a relative lack of stability leading to drug leakage. New hybrid nanoparticles have recently been developed to combine the respective advantages of the different subfamilies, namely solid-lipid, hybrid polymer-lipid [18] and hybrid organic-inorganic nanoparticles [19].

Nanoparticular vectorization is traditionally believed to take advantage of the enhanced permeability and retention (EPR) effect that results from the abnormal tumor vasculature causing preferential extravasation and increased concentration of nanoparticles in tumors [9, 20, 21]. Recent evidence also supports the existence of an additional active uptake process through endothelial cells [22]. However, even though the global biodistribution of nanoparticles seems to rely mostly on these mechanisms, only actively targeted nanoparticles efficiently infiltrate tumors and enter malignant cells [2, 23]. This requires coupling nanoparticles to targeting molecules – directed against surface antigens overexpressed on tumor cells – including but not limited to proteins (e.g. antibodies [24, 25]), aptamers [26], peptides [27] or polysaccharides [28]. An emerging alternative modality of active tumor targeting is the external magnetic guidance of metallic nanoparticles to promote preferential tumor extravasation [29]. Their coupling to iRGD peptides – recognized by the α_v_β_3_ integrin overexpressed on both the tumor neovasculature and some malignant cells – was also reported to improve the specific extravasation of nanoparticles in tumors [23, 27].

**Fig. 5:**
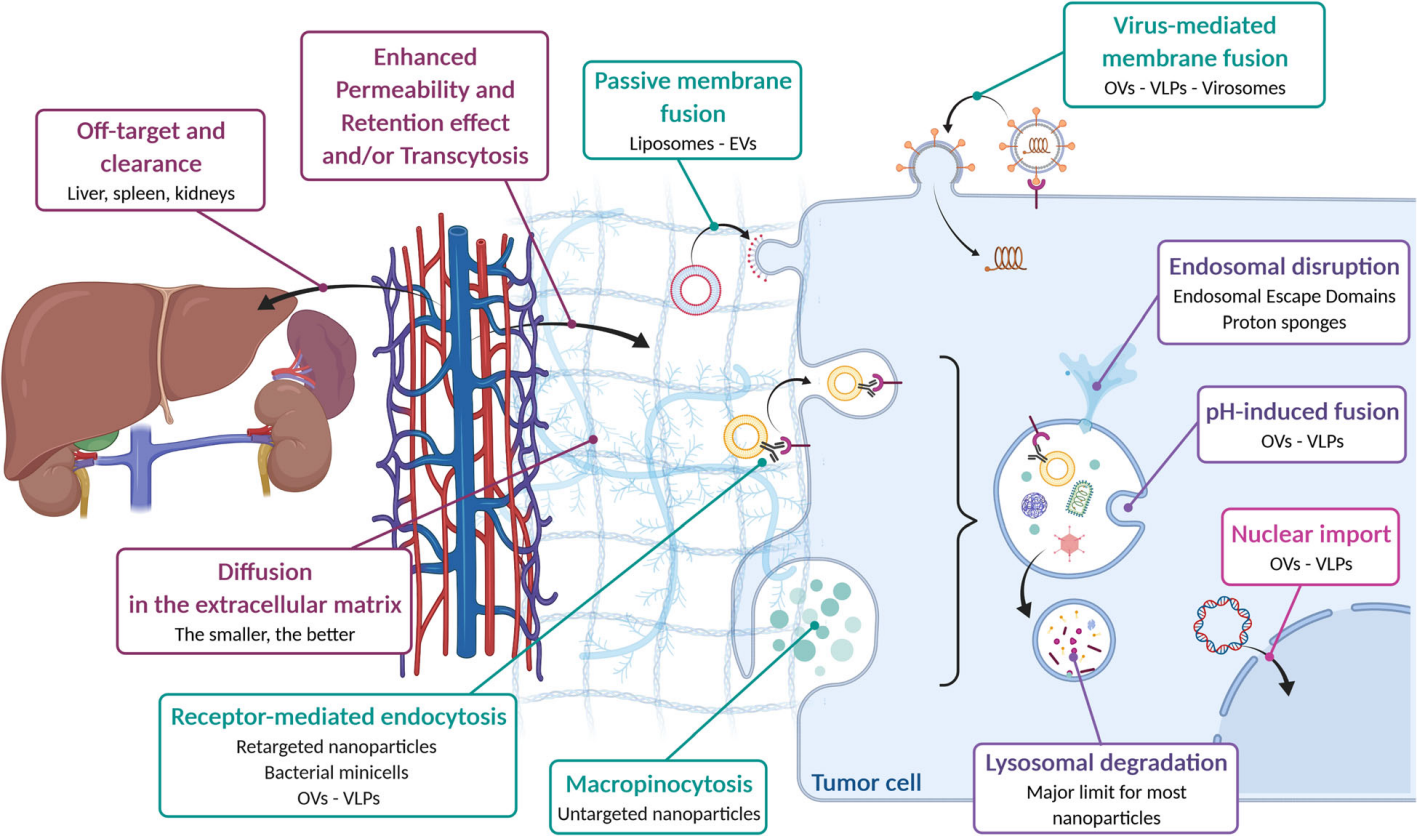
From the blood to the tumor cell: the difficult journey of nanovectors. Systemically injected nanovectors face several biological barriers to reach the tumor microenvironment and exert their therapeutic effect in malignant cells. First, filtering organs such as the liver (for nanovectors > 5 nm) or the kidneys (for nanovectors < 5 nm) eliminate an important fraction of the injected nanovectors. Nanovectors then extravasate from the bloodstream to the tumor either because of an increased vascular permeability (Enhanced Permeability and Retention effect) or by active transcytosis through endothelial cells. The nanovectors have to overcome the interstitial pressure and to diffuse in the extracellular matrix to reach tumor cells. This can be partially improved by active targeting strategies through nanovector engineering. Once reaching the cancer cells, nanovectors can be internalized by several mechanisms (e.g. passive or virus-mediated fusion, endocytosis, macropinocytosis) depending on their origin, size, composition and functionalization. The final difficulty consists in delivering the therapeutic cargo in the appropriate cellular compartment – generally the cytoplasm – to achieve optimal therapeutic efficacy. This usually requires further vector engineering (e.g. endosomal escape domains, pH-sensitive moieties), in particular for non-biological nanoparticles.EVs: Extracellular Vesicles; VLPs: Virus-Like Particles; OVs: Oncolytic Viruses

Overall, nanoparticles act as multimodal platforms that can be extensively engineered to improve both tumor targeting and the delivery of combined treatments to malignant cells; they are perfectly suited to increase both the half-life of therapeutic molecules in the bloodstream and their concentration in tumors while lowering their systemic toxicity [3]. Nevertheless, they face several biological barriers that have limited their clinical use so far (Fig. 5). These hurdles can however be overcome by rational engineering [3, 9]. As such, clearance by the mononuclear phagocytic system is usually diminished by functionalizing nanoparticles with non-immunogenic hydrophilic polymers such as polyethylene glycol (PEG) or zwitterionic ligands [30]; this prevents interactions with immune cells – thereby enhancing their half-life in blood – but can also decrease internalization by tumor cells. Of note, PEG can also be recognized by-anti-PEG antibodies that will impair vectorization efficacy and may generate immune-related adverse effects [31]. To improve the cellular intake of PEGylated nanoparticles within tumors, stealth polymer coatings that specifically dissolve in the tumor microenvironment (TME) have been developed [32]. Stealthiness can also be improved by entrapping nanoparticles into cellular membranes to mimic biological vesicles [19]. A lot of work has been performed lately to study the effect of the protein corona formation around nanoparticles, as it can drastically impact their stealthiness and tumor uptake [33–35]. Tunable drug release solutions have also been created to promote a specific delivery of packaged drugs exclusively in tumors. Hence, so-called ‘smart’ drug delivery systems enclose pH-, enzyme-, heat- or photo-sensitive molecules which conformations change in tumors to specifically destabilize the nanoparticle structure and release the therapeutic cargo [9, 36]. To improve nanoparticle tissue penetration and diffusion through the dense extracellular matrix (ECM) in tumors, several combinations of ECM-modifying molecules and nanoparticles are also currently under investigation [37]. Finally, a major pitfall for vectorization with nanoparticles is their trapping in endo-lysosomes after endocytosis, which exposes the therapeutic cargo to degradation. Available solutions include coupling nanoparticles to endosomal escape domains or proton sponges to destabilize endosomes and promote drug release toward the cytoplasm [38].

### Biological and bio-inspired nanovesicles

The biological world provides attractive alternatives to artificial lipid-based nanoparticles. Extracellular vesicles (EVs) are naturally occurring vesicles produced by eukaryotic cells and play important roles in intercellular communications [39]. They naturally package a broad range of cargos, from nucleic acids to proteins or lipids. There are two main types of EVs at the nanometer scale, namely microvesicles (50 nm to 1 μm) and exosomes (50 to 150 nm) that differ by their biogenesis and composition. Microvesicles directly bud outward of the plasma membrane while exosomes are generated from the inward budding of endosomal membranes and are released in the extracellular environment by exocytosis (Fig. 4). Because of their low immunogenicity and their efficient intake by cells [40], EVs have been investigated as drug nanocarriers for cancer therapy [41]. Therapeutic drugs can be loaded either directly into pre-formed vesicles or through modification of the EV-producing cells (e.g. drug exposure, transfection) to entrap the cargo into EVs during their formation [42, 43]. Although still controversial [44], EVs are suspected to possess inherent targeting capacities depending on their progenitor cell type [45]; tumor cell-derived exosomes thus appear to preferentially home to their cell types of origin *in vitro* compared with untargeted liposomes [46]. As for liposomes, the surface of EVs can be modified with targeting molecules or PEG [47]. Nevertheless, the lack of content standardization and of large-scale production methods still hinders their clinical use; the development of EV-like nanovesicles, which are basically liposomes enriched with membrane proteins to enhance cellular intake, is expected to help overcoming some of these limitations [48]. A derivative from this idea are “virosomes” (150 to 500 nm) that are composed of a synthetic lipid bilayer containing viral or parasitic fusogenic glycoproteins [49, 50]. Those take advantage of the ability of viral envelopes to recognize the targeted cells and to promote direct fusion with the plasma membrane, hence skipping the potential degradation of the encapsulated cargo into late endosomes after endocytosis (Fig. 5). Other strategies use cell-derived nanovesicles to camouflage other types of vectors (e.g. nanoparticles, viruses) to take advantage of their intrinsic properties and to escape neutralizing antibodies [51–54].

The trend to exploit bio-derived nanostructures for cancer therapy extends to different families of pathogens. Bacterial minicells (200 to 400 nm) are achromosomal vesicles produced by bacteria upon ectopic septation [55] (Fig. 4), an asymmetric division obtained by deleting the Min operon [56]. Minicells can be produced from Gram-positive and Gram-negative bacteria and contain all the molecular components of the parent cell except for the chromosome. Because of their vesicular structure, they are an alternative to lipid-based nanoparticles for cancer therapy (Fig. 3). Although Gram-positive minicells are negative for lipopolysaccharides (LPS) and may be ultimately more adapted for clinical use, most studies have used Gram-negative minicells that can be easily redirected to cancer-specific receptors (e.g. HER2/neu) with bispecific antibodies targeting both the LPS O-antigen on minicells and a tumor marker [57, 58]. Bacterial minicells can package a wide variety of molecules with different structures, charges and solubilities in an easier way than with lipid-based nanoparticles [55, 59]. They display a high loading capacity – up to 1,000 times higher than liposomes – following simple drug importation through the outer membrane via the non-specific FadL or OmpW channels. To confirm their interest in cancer therapy [60, 61], comprehensive studies are still needed to better characterize their properties, among which their immunogenic profile. Their safety however pleads for further developments, as was demonstrated in three recent phase I clinical trials that tested Epidermal Growth Factor Receptor (EGFR)-targeted minicells loaded with either paclitaxel [62], doxorubicin [63] or miRNA mimics [64] in patients with end-stage solid cancers, glioblastoma or mesothelioma, respectively.

### Virus-like particles

Viruses are extensively studied in therapeutic vectorization due to their active cell entry mechanisms, biocompatibility and well-characterized structures. Virus-like particles (VLPs) were developed to mimic animal, plant or bacteria viruses without retaining the ability to replicate in human cells [65] (Figs. 3 and 4). They are viral capsids with an icosahedral or filamentous structure composed of self-assembled proteins. Their diameters range from 25 (e.g. *parvoviridae*) to several hundred (e.g. *herpesviridae*) nanometers and they can contain a non-infectious genome composed of single- or double-stranded RNA or DNA [66]. Icosahedral VLPs can be used as genome-free particles such as the ones derived from the MS2 bacteriophage [67], which spontaneously assemble during protein production in bacteria, or from the cowpea mosaic virus (CPMV) [68]. On the contrary, filamentous VLPs derived from plant viruses and bacteriophages generally require a template genome for capsid proteins to assemble around it and form a rigid or flexible tube which length and width are determined by the capsid protein and the genome size. In addition, some viruses (e.g. *retroviridae*) present an envelope composed of an external lipidic membrane acquired while budding from the host cell surface [69]. As VLPs contain non-self proteins and potential pathogen-associated molecular patterns, they can be immunogenic and were mostly assessed as anti-cancer immuno-stimulatory treatments [70]. Their use as vaccines showed a good safety profile that makes them suitable for future use as nanovectors. Nevertheless, repeated treatments could promote the generation of antibodies and clearance by immune cells resulting in decreased tumor delivery. Capsid PEGylation or elimination of immuno-dominant epitopes can however limit these issues [71].

Because of their viral nature, VLPs are perfectly adapted to the delivery of therapeutic nucleic acids [72] but empty capsids can also be modified to transport other types of molecules. As such, the fixed structures of VLPs allow for extensive genetic and chemical engineering. Examples include tobacco mosaic virus VLPs that can be loaded by simple infusion and ionic interactions with their inner surface [73], the hepatitis B virus capsid that can be disassembled and re-assembled to capture a compound [74], or the functionalization of MS2 VLPs by inserting genetically a cystein residue in the capsid [75]. Interestingly, filamentous VLPs show a natural biodistribution to tumors after systemic injection, which could be mediated by their physical behavior in the tumor microvasculature [76, 77]. Non-human virus-based VLPs did not evolve to recognize human cell receptors; they produce less off-target effects but require genetic or chemical retargeting to malignant cells. Common modifications involve the retargeting of VLPs with cancer-specific peptides [78], aptamers [75] or other molecules [72, 79], or the pseudotyping of enveloped VLPs with exogenous proteins. Similarly, twelve serotypes of adeno-associated viruses (AAVs) have been identified so far [80] and could be used to target different types of cancers. In addition, VLPs from plant or bacteria viruses cannot easily escape human endo-lysosomes and display lower transfer efficacy, even after retargeting [81–84]. Strategies similar to the ones used with nanoparticles for endosomal escape and cargo delivery are being tested to overcome these limitations [78]. On the opposite, VLPs derived from human pathogens benefit from coevolution to achieve efficient gene transfer inside human cancer cells (Fig. 5).

### Oncolytic viruses

Contrary to VLPs for which the non-replicative nature is a major determinant of their clinical safety and intermediate immunogenicity, oncolytic viruses (OVs) display all the properties of natural viruses except that their replication is restricted to malignant cells [85] (Fig. 4). The diversity of OVs has been reviewed extensively elsewhere [86] and is summarized in Fig. 3. OVs are either naturally attenuated viral strains or genetically engineered viruses that harness cancer hallmarks such as altered metabolism, immunosuppression or resistance to cell death that make tumors more sensitive than healthy tissues to viral infections. Tumor cells also commonly overexpress surface proteins that are used by some viruses for cell entry [87, 88]. For many oncolytic RNA viruses, tumor specificity mainly depends on defects in the innate antiviral pathways commonly acquired by malignant cells during tumor evolution [89, 90], while DNA viruses can be modified with tumor-specific promoters [91]. Contrary to other nanovectors, the tumor specificity of OVs thus mostly relies on post-entry restriction rather than selective entry through specific surface markers. They also exhibit therapeutic properties on their own as they can both directly kill tumor cells and activate a diversity of immune cell types involved in the anti-tumor responses [86, 92]. After two decades, more than a hundred trials and few regulatory approvals for clinical use [93–95], they have demonstrated a very good safety profile but a somewhat modest therapeutic efficacy in humans.

To improve their intrinsic anti-cancer properties, OVs are commonly armed to vectorize therapeutic transgenes that will be expressed by infected malignant cells in the TME, thereby making them *bona fide* nanovectors [96]. Viruses have evolved to deliver efficiently their genome in host cells and are thus perfectly designed to vectorize nucleic acids (Fig. 5). The first OV to be approved by the US and EU regulatory agencies in 2015 was the recombinant herpesvirus Talimogene laherparepvec (T-VEC) that encodes the Granulocyte-Macrophage Colony-Stimulating Factor to enhance its immunostimulatory properties [94, 97]. The transgene capacity of viruses is however limited by the fitness cost – the longer the genome, the longer it takes to replicate – and the size limit of the viral particle; DNA viruses generally exhibit a higher transgene capacity than RNA viruses. OV replication capacity allows both spreading of the transgene in the tumor and its sustained expression over time [98]. As with VLPs, surface molecular coupling is theoretically possible – especially for non-enveloped viruses – to enable intracellular delivery of drugs in specific cells.

The current standard for OV treatment is intratumoral injection with the limit that only reachable tumors can be treated, but recent evidence of viral replication in tumors following intravenous administration in patients have been reported [99–103]. Despite pre-existing immunity having no measurable effect on the therapeutic outcome after intratumoral injection, innate and adaptive immune responses against circulating viruses may restrict their efficacy after intravenous administration [104, 105]. PEGylation of OVs [106, 107] or switching OV species during the course of treatment [108, 109] can improve stealthiness and enhance treatment efficacy. Enveloped viruses can also be pseudotyped with different viral envelops [110–112], while changing the serotype of non-enveloped viruses could evade the immune response [113–115]. Finally, the titration of OVs by healthy cells after non-specific entry – distinct from their tumor-specific replication and killing – can be answered by retargeting OVs to tumor-specific surface antigens through genetic engineering. Advances made in the field of nanoparticles for chemical modifications are also expected to lead to alternative solutions [107].

## Applications in cancer therapy

### Chemotherapy

Cancer chemotherapeutics are a large family of chemical drugs [116] that affect highly proliferating malignant cells and exhibit diverse modes of action from cell cycle arrest to cell death induction and epigenetic modulation. These molecules often lack tumor specificity and healthy proliferative cells are frequently impacted, thereby causing different debilitating symptoms. Consequently, vectorization of chemotherapeutics is critical to improve their tumor specificity and diminish side toxicities. Here, we present an overview of how the different families of nanovectors can help bypassing the major limitations of chemotherapies, including their poor aqueous solubility, their lack of tumor specificity and the acquisition of resistances. The advantageous physical properties of some nanovectors that can be exploited in combinatorial strategies with chemotherapies are also discussed.

#### Solving drug insolubility

Chemical drugs for cancer treatment vary widely by their structures, charges and solubilities that can limit their clinical use, an illustrative example being the high hydrophobicity of taxanes [117]. The nanomedicine field however provides numerous solutions for drug vectorization whether they are hydrophobic (e.g. paclitaxel, cisplatin) or amphipathic (e.g. doxorubicin, 5-fluorouracil). As explained above, the diversity of chemically engineered nanoparticles with variable loading and functionalization possibilities makes them the most suitable for vectorizing chemotherapeutic drugs [9, 118] (Table 2). Hydrophilic drugs can be easily encapsulated inside liposomes, adsorbed in pores of silica nanoparticles or conjugated on metallic or polymeric nanoparticles using reactive hydroxyl, carboxyl, amino or thiol groups. Hydrophobic molecules are commonly loaded in micelles or solid-lipid nanoparticles or inserted in the lipid bilayer of liposomes. Nanoparticles are also used to vectorize hydrophobic epigenetic modulators (e.g. inhibitors of histone deacetylases or DNA methyltransferases) to improve their pharmacokinetics and therapeutic efficacy [119–122]. Macromolecular nanoassemblies and lipid-based nanoparticles have been used to vectorize almost all types of chemotherapeutics and several nanomedications have either already been approved by the FDA for cancer treatment or are currently evaluated in clinical trials [8, 123] (Table 3). It is interesting to note that cancers with very different profiles, from end-stage solid tumors to hematological malignancies, can be eligible to nanovectorization of chemotherapeutics. As an example, the nab-paclitaxel formulation (Abraxane®) – composed of paclitaxel fused to human albumin nanoparticles – has demonstrated improved safety and efficacy compared to free paclitaxel [136] and is approved against non-small cell lung cancer, metastatic pancreatic cancer and as a second-line treatment for metastatic breast cancers [137].

**Table 3:** Representative examples of the advancement of nanovectors in cancer therapy

Nanovector family	Therapy	Drug administration	Phase	Cancer types	Route of administration	References
*Organic nanoparticles*	*Chemotherapy*	PEGylated liposomal doxorubicin (Doxil®/Caelyx®)	Approved (1995)	Ovary, Kaposi’s sarcoma, multiple myeloma	Intravenous	[137]
		Non-PEGylated liposomal doxorubicin (Myocet®)	Approved (2000)	Breast	Intravenous	
		Albumin particle-bound paclitaxel (Abraxane®)	Approved (2005)	NSCLC, breast, pancreas	Intravenous	
		PEGylated liposomal irinotecan (Onivyde®/MM-398®)	Approved (2015)	Pancreas	Intravenous	
		Non-PEGylated liposomal cytarabine:daunorubicin (VYXEOS®/CPX-351®)	Approved (2017)	AML	Intravenous	
	*Gene therapy*	TR-targeted liposomes encapsulating a p53-encoding plasmid (SGT-53®)	I/II	Pediatric solid tumors, glioblastoma, pancreas	Intravenous	NCT02354547, NCT02340117, NCT02340156
	*RNA interference*	Lipid nanoparticles encapsulating interfering RNAs	I/II	Solid tumors, Edwing’s sarcoma, liver, AML	Intravenous	[247]
	*TME modification*	Various NPs for CAFs, TAMs, ECs, ECM suppression or normalization	Preclinical	Various cancer models	Mostly intravenous	[291]
	*Immunotherapy*	Vectorization of various immunomodulators	Preclinical	Various cancer models	Mostly intravenous	[291]
*Inorganic nanoparticles*	*Hyperthermia*	Minosilane-coated iron oxide nanoparticles (Nanotherm®)	Approved (2010)	Glioblastoma	Intratumoral	[307]
	*Radiotherapy*	Hafnium oxide nanoparticles (NBTXR3®/Hensify®)	Approved (2019)	Squamous cell carcinoma	Intratumoral	[137]
	*RNA interference*	siRNAs adsorbed on gold nanoparticles	I	Glioblastoma	Intravenous	[247]
*Bacterial minicells*	*Chemotherapy*	EGFR-targeted, doxorubicin-loaded minicells	I/II	Glioblastoma	Intravenous	[63]
	*RNA interference*	EGFR-targeted minicells containing a miRNA mimics cocktail	I	Mesothelioma, NSCLC	Intravenous	[64]
*Extracellular vesicles*	*Chemotherapy*	Tumor-derived microvesicles packaging methotrexate	II	Lung cancer	Intravenous	NCT02657460
	*Gene therapy*	Tumor-derived exosomes loaded with CRISPR-Cas9 against PARP1	Proof-of-concept	Heterotopic ovarian cancer model	Intravenous	[308]
	*RNA interference*	MSC-derived exosomes loaded with anti-KrasG12D siRNAs	I	Metastatic prostate cancer	Intravenous	NCT03608631
*Virus-like particles*	*Chemotherapy*	Tobacco Mosaic Virus carrying phenanthriplatin	Preclinical	Heterotopic breast cancer model	Intravenous	[73]
	*Gene therapy*	TP53-encoding non-replicating adenovirus	Diverse	Solid cancers	Mostly intratumoral	[208]
		M13 phage encoding HSV-TK	Preclinical	Orthotopic glioblastoma model	Intravenous	[309]
	*RNA delivery*	MS2-derived VLPs carrying siRNAs	Proof-of-concept	Hepatocellular carcinoma cell line	NA	[310]
*Oncolytic viruses*	*Chemotherapy*	HSV-TK-encoding adenovirus	II	Triple-negative breast cancer, NSCLC, prostate	Intratumoral	NCT03004183, [311]
		HSV-TK-encoding vaccinia virus	II	Solid tumors	Intravenous	NCT04226066
	*Radiotherapy*	NIS-encoding measles virus	II II	Multiple myeloma Ovarian, fallopian and peritoneal cancers	Intravenous Intraperitoneal	NCT02192775 NCT02364713
	*Gene therapy*	TP53-encoding replicating viruses	Preclinical	Many solid cancers models	Intravenous / Intratumoral	[208]
	*RNA interference*	Oncogene silencing with small RNAs-encoding Adenovirus and HSV	Preclinical	Many solid cancer models	NA	[312, 313]
	*TME modification*	Hyaluronidase-expressing adenovirus	Preclinical	Orthotopic glioblastoma model	Intratumoral	[134]
	*Immunotherapy*	GM-CSF-encoding herpes simplex virus (Talimogene laherparepvec)	Approved (2015)	Melanoma	Intratumoral	[94, 97]

*AML* acute myeloid leukemia, *CAF* cancer-associated fibroblast, *EC* endothelial cell, *ECM* extracellular matrix, *EGFR* epidermal growth factor receptor, *HSV-TK* herpesvirus thymidine kinase, *NP* nanoparticle, *NSCLC* non-small cell lung carcinoma, *TAM* tumor-associated macrophage, *TR* transferrin receptor, *VLP* virus-like particle

Other types of nanovectors are currently studied to transport and deliver chemical drugs to tumors (Table 2). The characterization of VLPs at the atomic level allows for precise chemical coupling strategies similar to the ones used for nanoparticles. For example, doxorubicin coupling to Physalis Mottle virus icosahedral VLPs [81] or to truncated hepatitis B virus core antigen (tHBcAg) VLPs [138] improved both its cellular uptake and cytotoxicity against malignant cells. Doxorubicin and mitoxantrone were also passively loaded into CPMV [139] and filamentous plant viruses VLPs [140–142] by exploiting for the latter the negative charges of the inner side of the particles. Simple dissociation/association of tHBcAg allows for passive dual loading of polyacrylic acid (PAA) along with doxorubicin that will be released at low pH when no longer retained by protonated PAA [79]. EVs on their part display similar vectorization abilities as liposomes. They were shown for instance to deliver doxorubicin [143] or paclitaxel [144] *in vitro* to breast or prostate cancer cells, respectively, or paclitaxel to lung cancer cells after systemic administration in mice [145]. Packaging of decitabine in erythro-magneto-hemagglutinin nanovesicles showed a specific delivery to prostate cancer xenografts under *in vivo* magnetic guidance and a significant tumor mass reduction at a lower dose than with free decitabine [146]. Among the bio-inspired nanovectors, bacterial minicells may be the more promising as they can incorporate a wide variety of chemotherapeutic agents without drug efflux up to several days [55]. Their encouraging early clinical results in two phase I clinical trials that used EGFR-targeted bacterial minicells containing either doxorubicin or paclitaxel to treat patients with advanced solid tumors [62, 63] however need to be confirmed.

#### Improving tumor specificity

The lack of tumor specificity for chemotherapies causes off-target effects and limits clinical efficacy by decreasing drug concentration in tumors. For instance, doxorubicin displays elevated hematological and cardiac toxicities as a free molecule [147]. It has been vectorized as early as the 1990s in the first FDA-approved nanodrug Doxil®, which is currently approved for the treatment of ovarian cancer, multiple myeloma, metastatic breast cancer and Kaposi’s sarcoma. Doxil® is composed of doxorubicin encapsulated in untargeted, PEGylated liposomes that enable a high concentration of doxorubicin in tumors correlated with a higher tolerability compared to free doxorubicin [148]. This formulation was followed by many other combinations of chemotherapeutic drugs with numerous types of nanoparticles [124]. As with the Doxil® liposomal formulation, their tumor specificity mostly relied on passive targeting due to destabilized tumor vasculature and the resultant EPR effect. Based on a similar idea, the natural tumor distribution of filamentous VLPs [77, 149] can also be exploited for this purpose; PEGylated Potato Virus X (PVX) VLPs passively loaded with doxorubicin were indeed shown to elicit a better control of breast cancer xenografts in immunodeficient mice than doxorubicin alone [140]. However, a combination of PVX and doxorubicin was more effective than doxorubicin-loaded PVX in an immunocompetent melanoma model [141], suggesting that VLPs elicit an adjuvant anti-tumor immune response that participates in the therapeutic effect and pleading for the use of immunocompetent animal models for future evaluations.

Current studies mostly focus on actively targeted nanodrug formulations to enhance interactions of the nanoparticles with malignant cells after having reached the TME [23, 24, 27]. Several strategies have demonstrated increased drug concentration in tumors and enhanced therapeutic efficacy compared with the corresponding free molecules or untargeted nanovectors [23, 150]. In a preclinical study, paclitaxel-loaded nanocapsules constituted of a lipid core surrounded by a surfactant were targeted to the altered tumor vascular endothelium with an iRGD peptide [151]. The authors demonstrated that the targeted nanoparticles concentrated in hepatic tumors, induced specific cytotoxicity and were better tolerated than non-targeted nanoparticles. Another recent study showed that hybrid solid-lipid nanoparticles decorated with folic acid can significantly increase the concentration of carboplatin and paclitaxel in tumors cells in a murine cervical cancer model [152]. EGFR-targeted, doxorubicin-containing bacterial minicells were demonstrated to rapidly locate in spontaneous gliomas in dogs, a tumor usually difficult to reach because of the blood-brain barrier [60]. Another approach for active tumor delivery is to target the hypoxic center and acidic microenvironment of tumors, in particular using the pH (low) insertion peptide (pHLIP) [153]. An example for this strategy is the use of doxorubicin-loaded bacterial minicells with a pHLIP added to their membrane, which successfully invaded the necrotic and hypoxic regions of orthotopic murine breast cancers and achieved a significant tumor reduction compared to both free drug and untargeted minicells [154].

#### Fighting resistance

Cancer cells commonly develop resistance against chemotherapies, for instance by acquiring a multidrug resistance (MDR) phenotype. This can result from the expression of ATP-dependent transporters that promote the efflux of drugs outside the cell to escape death induction [155, 156]. Nanovectors enable drug immobilization and limit efflux, thereby enhancing drug concentration in tumor cells. They can also carry several drugs at the same time to strike cancer cells on different fronts simultaneously and prevent therapeutic escape [157]. Such strategies can combine several chemotherapies [152] or different types of treatments such as a combination of a chemotherapeutic drug with a siRNA [158]. Doxorubicin-coated, multifunctional mesoporous silica nanoparticles containing a siRNA against the P-glycoprotein (Pgp) drug exporter showed targeted Pgp knockdown and a synergistic inhibition of resistant breast tumor growth in preclinical models [159]. A similar approach used sequentially (i) CD33- or EGFR-targeted bacterial minicells containing a plasmid coding for shRNAs against MDR pumps and (ii) chemotherapies [160]; mice bearing drug-resistant colorectal, breast or uterine tumors were efficiently treated without toxicity as a thousand-fold less drug and shRNA were used compared to conventional systemic treatment. Another way to circumvent tumor resistance is to use highly cytotoxic compounds – such as the PNU-159682 metabolite [161] – that cannot be injected systemically because of their high toxicity. Systemic vectorization of this drug in EGFR-targeted bacterial minicells showed significant tumor reduction and immune activation with no side effects in immunocompetent breast and colorectal murine models but also lung and colorectal human cancer xenografts [162].

#### Exploiting intrinsic physical properties

Some chemically engineered nanoparticle families have intrinsic physical properties that make them suitable for combined therapies. As such, gold nanoparticles can be used for photothermal therapy, which consists in a local vibrational heat generation through the absorption of specific wavelengths of light [163]. Super Paramagnetic Iron Nanoparticles (SPIONs) on the other hand can be used for hyperthermia, a local heat generation under a magnetic field [164]. Those two phenomena have demonstrated a moderate therapeutic efficacy on their own but can sensitize cancer cells to chemotherapies loaded in the same nanoparticles [165]. Indeed, hyperthermia and photothermia inhibit the repair of DNA lesions (e.g. double-strand breaks) generated by chemotherapy or radiotherapy [166]. Several clinical trials involving the use of hyperthermia as adjuvant for chemotherapy are ongoing [167]. An example is the use of a near-infrared-responsive polypeptide nanocomposites charged with doxorubicin and capable of heat generation and heat-sensitive nitric oxide (NO) gas delivery [168]. This combination of photothermia, NO gas therapy and chemotherapy achieved complete breast tumor regression in mice after a single near-infrared irradiation. Hyperthermia can also be used to release chemotherapeutics enclosed in hybrid delivery systems constituted of nanoparticles associated with thermosensitive molecules [169]. Regarding epigenetic modulation, some studies suggest that metallic and silica nanoparticles could directly induce modifications of DNA methylation or of histone acetylation and disrupt miRNA expression [170, 171], but the significance of these modifications in the context of cancer treatment is still to be investigated.

The nanovectorization of chemotherapeutic drugs has been historically dominated by the use of organic nanoparticles (Table 2), supported by their unmatched diversity of structures and compositions (Fig. 2). This led to different clinical successes resulting in several drug approvals (Table 3). However, the more recent advances in vesicular nanovectors (e.g. bacterial minicells, EVs), provide new solutions with enhanced biocompatibility (Table 1) that may advantageously replace synthetic nanoparticles in some clinical contexts. Studies on VLPs are at an earlier stage of development but also demonstrated interesting properties in preclinical experiments. In the end, hybrid vectorization systems incorporating both synthetic and biological moieties may constitute a rational compromise between efficacy, biocompatibility and standardized manufacturing even if complex designs may generate additional difficulties for clinical development.

### Radiotherapy

Half the cancer patients receive radiotherapy – which exploits the low resistance of tumor cells to radiation-induced DNA damages – during their course of treatment [172]. Overexposure of healthy cells to radiations leads to radiotherapy-related toxicities that could be partially addressed using appropriate vectorization strategies. For external-beam radiotherapy [173] – or for related photodynamic therapy (PDT) that uses non-ionizing wavelengths [163] – nanovectors can sensitize tumors to radiations. For internal radiotherapy, nanomedicine is an elegant solution to deliver specifically radioelements to tumors and an alternative to the use of radiolabeled antibodies in radioimmunotherapy approaches [174].

- ***Radiosensitization***

Radiations not only cause direct damages to biomolecules but also generate reactive oxygen species (ROS). This phenomenon can be enhanced in tumors by the vectorization of radiosensitizing molecules that increase either ROS production in response to ionizing beams or malignant cell sensitivity to both direct and indirect radiation effects [175]. Gold nanoparticles (AuNPs) are well-characterized for their radiosensitizing properties [176]; their concentration in tumors increases the dose delivered locally during radiotherapy, resulting in ROS production, DNA repair machinery impairment and improved treatment efficacy. However, the clinical translation of these metallic nanoparticles remains challenging because of both their tendency to aggregate after systemic injection and their long-term toxicity due to liver accumulation. An alternative are chemical ROS-generating photosensitizers that can be coupled to a wide variety of biocompatible nanoparticles for PDT [177, 178]. Interestingly, some chemical radiosensitizers are also able to self-assemble to generate nanostructures by themselves [179]. Upconverting nanoparticles were recently modified to assemble with a photosensitizer *in vivo* by click chemistry after systemic injection [180]. These nanoparticles are able to convert low energy near-infrared light into high energy photons that activate the photosensitizer to generate ROS and achieved inhibition of tumor growth in an ectopic breast cancer model. A recent study used EVs purified from mouse blood and surface-loaded with the photosensitizer protoporphyrin IX (PplX) in a two-stage irradiation protocol to efficiently deliver PplX and induce apoptosis by PDT in a breast tumor model [181]. The porphyrin photosensitizer has also been effectively vectorized with M13 filamentous phage VLPs retargeted to mammary cancer cells by a specific peptide displayed on the pVIII coat protein and demonstrated efficient cancer cell targeting and sensitization to PDT [182]. The lack of oxygen in the tumor hypoxic core can lead to radioresistance, which can be bypassed by developing nanoparticles with O_2_-elevating abilities or nano-radiosensitizers with diminished oxygen dependence [183]. As an example, mesoporous manganese dioxide nanoparticles are able to catalyze O_2_ production to actively reverse hypoxia in tumors. These nanoparticles were loaded with the photosensitizer acridin orange and exhibited enhanced radiotherapy efficacy both *in vitro* and *in vivo* in a lung cancer xenograft model [184]. Hypoxia-reverting liposomes [185], macromolecular nanoassemblies [186, 187] and other types of nanoparticles [177] have also been used for their photosensitizing properties.

The radiosensitizer family also encompasses all molecules able to enhance tumor cell sensitivity to radiation effects by interfering with essential cellular pathways like DNA repair, apoptosis induction or cell cycle progression. As such, chemotherapeutics are used as radiosensitizers at the clinical level [175] and their loading on chemically engineered nanoparticles have demonstrated radiosensitizing effects [185, 188, 189]. As for chemotherapy, SPIONs and gold nanoparticles alone or within a bigger organic nanoparticle can also mediate tumor radiosensitization through inhibition of DNA repair mechanisms by hyperthermia or photothermia, respectively [166]. DNA viruses are capable of impairing the DNA damage response [190] and some OVs (e.g. *adenoviridae*) naturally downregulate key proteins involved in the response to radiation-induced DNA damages [191], which makes them intrinsically radiosensitizing [192]. SiRNA-mediated gene silencing is another strategy to target genes involved in the cellular response to ionizing radiations [175]. As discussed below, OVs and VLPs are useful tools for such small RNA vectorization, an example being an adenovirus encoding a shRNA against the DNA-dependent protein kinase DNA damage response protein for local enhancement of radiotherapy in a human colorectal cancer xenograft model [193].

b. ***Internal radiotherapy***

Radionuclides have been vectorized for several years with various nanovectors like VLPs [194–196], EVs [197], nanoparticles [198, 199] or an oncolytic adenovirus [200] for cancer imaging, but for VLPs or EVs this has yet to be studied in therapeutic protocols. High-energy, short-range alpha-emitters have been conjugated to various types of chemically engineered nanoparticles with good therapeutic results but a large majority of radionuclides currently used in cancer therapy are low-energy beta-emitters with a longer path length [201]. Iodine 131 ( [143]I) is the most common nanoparticle-coupled radionuclide reported in the literature. Recent examples include PEGylated, nuclei-targeted [143]I-AuNPs tested in a colorectal cancer model [202] and [143]I-labeled, human serum albumin-bound manganese dioxide nanoparticles that were capable of significantly inhibiting tumor growth in a breast cancer model with a potentiating effect of MnO_2_ on radiotherapy efficacy [203]. In another study, treatment with PEGylated liposomes enclosing an [143]I-albumin core led to subcutaneous breast tumor shrinkage when co-administered either with liposomes containing a photosensitizer or with an anti-PD-L1 antibody [204]. In a very different strategy, OVs coding for the human sodium-iodine symporter (NIS) have been used to enhance the specific intake of [143]I in OV-infected tumor cells [99, 205–207]; OV-NIS are injected several days before [143]I and indirectly mediate the vectorization of the radioelement to tumors neo-expressing NIS.

As for chemotherapy, the different subfamilies of nanoparticles have been massively investigated to improve the efficacy of radiotherapy, but the low biocompatibility and biodegradability of inorganic nanoparticles called for the development of alternatives. Successful delivery of radiosensitizing molecules was achieved with organic nanoparticles and bio-inspired vectors such as EVs, while engineered VLPs can be chemically coupled to radionuclides. Viruses and other bio-derived vectors are also expected to define original approaches to exploit precise biological mechanisms that are involved for instance in the cellular response to radiations.

### Delivery of nucleic acids

Malignant transformation results from gene alterations (e.g. deletions, amplifications, mutations, translocations, epigenetic or viral dysregulations) that displace the equilibrium between oncogene and tumor-suppressor gene expression. These alterations can be corrected or compensated using nucleic acids (DNA or RNA) for gene editing (over-expression or knock-out), direct induction of cell death by expression of toxic genes or by modulating gene expression. As free nucleic acids are rapidly degraded in the bloodstream and do not cross cell membranes, clinical translation of cancer gene therapy requires proper vectorization [208]. Viruses are particularly suited for this as they are naturally designed to deliver genes in targeted cells (Table 2). Transgenic viruses are also relatively simple to generate and they ensure a high level of transgene expression. Many studies were conducted with retrovirus-like particles (RLPs) [209], non-replicative adenoviruses [210] and AAVs [211], whereas other VLPs used for both their capacity to package DNA and their easy retargeting achieved lower transduction efficacy [68, 82, 212]. Despite several limitations – the main one being the cytoplasmic delivery of cargos initially addressed to the nucleus – nanoparticles (mainly lipid-based) have been extensively used for nucleic acid delivery [213–215]. Some strategies are developed to increase nanoparticle-mediated gene expression in tumor cells [216], for instance by using nuclear localization signals (NLS) or by vectorizing messenger RNAs [217].

#### Gene therapy

The most frequent genetic alterations in cancer being p53 mutations, most gene therapies consist in vectorizing a wild-type *TP53*. Restoring wild-type p53 functions triggers cell death specifically in highly-dividing tumor cells exhibiting genome instability. An example of a nanovector exploiting this mechanism is Gendicin, a p53-encoding adenoviral vector that was the first-in-class gene therapy treatment for head and neck cancer approved by the China Food and Drug Administration in 2003 [218]. While many years of clinical use demonstrated its safety, its efficacy remains limited. However, the co-vectorization of other tumor suppressors (e.g. *ING4, PTEN*) in the same vector demonstrated synergistic efficacy [83]. The enhanced vectorization potential and intrinsic tumor cytotoxicity of OVs were also exploited to transiently express tumor suppressors at high levels but still lack clinical assessment [129]. Regarding nanoparticles, liposomes containing p53-encoding plasmids are being evaluated against different types of solid cancers [219, 220], including in phase I/II clinical trials (NCT02354547, NCT02340156, NCT02340117).

Other studies focus on cancer gene editing to disable key oncogenes. An oncolytic myxoma virus carrying a CRISPR cassette targeting the *NRAS* oncogene demonstrated efficient gene editing *in vivo* along with prolonged survival in a xenograft model of rhabdomyosarcoma [221]. Similarly, a CRISPR-Cas12a-carrying oncolytic adenovirus efficiently edited *EGFR in vivo* specifically in xenografted lung adenocarcinoma cells [222]. Transgene-free retroviral VLPs loaded with Cas9-sgRNA ribonucleoproteins (“nanoblades”) that demonstrated *in vivo* genome editing capacity [223] and can be pseudotyped to modulate their cell tropism may also have interesting applications for cancer therapy. Alternatively, lipid-based nanoparticles [224] and macromolecular nanoassemblies [225, 226] have been successfully used to deliver CRISPR-Cas9-encoding plasmids for oncogene edition. As an example, tumor-targeted macromolecular nanoassemblies decorated with a NLS-containing peptide specifically delivered a CRISPR-Cas9 plasmid to the nuclei of lung cancer cells *in vitro* and efficiently knocked out the *Catenin beta-1* gene [227]. Nevertheless, the dysregulation of tumor suppressor genes in cancer being frequently post-transcriptomic, this may limit the actual efficacy of gene editing. In addition, gene delivery mostly impacts the cells receiving the transgene and will have limited bystander effects. Other approaches may thus be more adapted to address the heterogeneity of malignant diseases.

#### Induction of cell death

Gene therapies for triggering specific tumor cell death include Gene-Directed Enzyme/Prodrug Therapy (GDEPT) [228] and cytotoxic gene therapy [229, 230]. GDEPT involves the tumor delivery of a transgene encoding an enzyme able to convert a non-toxic prodrug into a cytotoxic drug, the latter exerting its activity against the modified tumor cells and its surrounding environment. Such transgenes include the herpes simplex virus thymidine kinase (*HSV-TK*) gene, converting ganciclovir into ganciclovir-triphosphate and inhibiting DNA elongation [231], and the cytidine deaminase that converts 5-fluorocytosine into 5-fluorouracile [228]. VLPs (e.g. adenoviruses) are the most suitable and the more frequently used nanovectors for suicide gene therapy due to their high gene transfer potential [232, 233]. For OVs, HSV *de facto* expresses HSV-TK [234] but this transgene has also been vectorized by other viruses [235, 236]. Liposomes were also used to actively deliver a mRNA or a plasmid coding for the HSV-TK protein in a lung cancer mouse model [237]. The authors showed that both mRNA- and plasmid-carrying liposomes can mediate a significant inhibition of tumor growth following ganciclovir injection with a superiority of the mRNA formulation. In another example, HSV-TK plasmid-bearing macromolecular nanoassemblies demonstrated a significant therapeutic effect against invasive orthotopic human glioblastoma multiforme in mice [238].

Cytotoxic gene therapy on the other hand consists in delivering a cell death-triggering gene to tumors. To avoid off-target effects, the expression is generally controlled by a cancer- or tissue-specific promoter [229]. The main focus has been on tumor necrosis factor (TNF)-related apoptosis-inducing ligand (TRAIL)-based cancer therapy, TNF-α and TRAIL being major mediators of death receptor-mediated apoptosis. Delivery of TNF-α- or TRAIL-encoding genes for secretion of the cognate proteins by tumor cells was reported with OVs [239], VLPs [82, 240] or nanoparticles [241] with evidence of a bystander effect. Interestingly, displaying the TRAIL protein on the surface of nanovectors has also demonstrated efficient TRAIL-mediated cell death induction of circulating tumor cells in different studies [242–244]. An alternative is the use of inducible suicide genes, an elegant example being the vectorization by adenoviral vectors [245] and AAVs [246] of the AP20187-dependent inducible version of caspase 9, activated after AP20187 treatment. Another example is the AAV vectorization of a CRISPR system targeting telomeres to induce tumor cell death [211]. Several pathogen-derived toxins have also been studied as cell death inducers for cancer cytotoxic gene therapy. An example is the tumor-specific, apoptosis-triggering viral protein apoptin that was encoded by lambda phage VLPs [247] or OVs [248] and induced significant tumor reduction in breast and lung cancer models, respectively. A recent innovative study described the design of macromolecular nanoassemblies loaded with a light-switchable transgene coding for the diphtheria toxin A inducible by blue laser light, a protocol that improved survival in a melanoma model [249]. In parallel to these gene delivery approaches, several groups also vectorized the different toxins as proteins to trigger selective cancer cell death with nanoparticles [250, 251], VLPs [131] and bacterial minicells [252].

#### Modulation of gene expression

Cellular pathways and gene expression can be precisely modulated by RNA interference (RNAi). This involves different types of small RNAs such as microRNAs (miRNAs) and small interfering RNAs (siRNAs) that interact with specific target mRNAs and stimulate their degradation or the inhibition of their translation [253]. The targeted inhibition of oncogenic mRNAs or miRNAs attracts attention but effective delivery of small RNAs for cancer treatment requires appropriate vectorization, in particular to reduce their degradation by nucleases. To date, siRNAs and miRNAs have been mostly vectorized by chemically engineered nanoparticles, in particular liposomes as extensively reviewed elsewhere [213, 254]. The safety of siRNA vectorization by liposomes – for instance against genes coding for the Ephrin type-A receptor 2 or B-cell lymphoma 2 (BCL-2) – is under evaluation in several ongoing clinical trials [125, 255]. SiRNAs directed against oncogenes (e.g. *MYC*, *BRAF*, *BCL-2*) have also been transported with macromolecular nanoassemblies or inorganic nanoparticles [256] and, more recently, an anti-survivin siRNA was efficiently vectorized with dendrimers that were further entrapped in tumor-derived EVs for treating mice bearing prostate carcinoma [54].

MiRNAs are naturally transported by EVs throughout the organism to modulate gene expression in neighboring or distant cells, both in physiological and pathological conditions [39]. This process was harnessed in several studies to deliver miRNAs or anti-miRNAs to cancer cells [47]. Human fibroblast-derived exosomes containing Kras^G12D^-targeted siRNAs were thus shown to mediate a better inhibition of tumor growth compared to liposomes in pancreatic cancer models [257]; this difference of efficacy was attributed to the lower immunogenicity and decreased clearance of exosomes. Similarly, mesenchymal stem cell-derived EVs were used to deliver several tumor-suppressing miRNAs to malignant cells by exploiting both their alleged natural tropism for tumors and immune evasion abilities [254, 258, 259]. Another example is the use of natural killer cell-derived exosomes loaded with a Let-7a miRNA-coupled dendrimer that were efficiently delivered *in vivo* to neuroblastoma cells [260]. However, the natural miRNA content of EVs may mediate unwanted effects in tumors and preclude clinical applications; one should carefully choose the EV donor cell type or opt for alternatives such as artificial exosome–mimetic nanoplatforms that simulate natural cell-derived exosomes but with a controlled composition [261]. Micro-organism-derived nanovectors are also a suitable alternative to vectorize miRNAs. In a phase I clinical trial, patients with malignant pleural mesothelioma were treated intravenously with EGFR-targeted bacterial minicells containing miRNA mimics [64]; the study concluded to treatment safety associated with a disease control rate of 65%, but the precise intake mechanism is still to be characterized. MS2 bacteriophage VLPs can be loaded with siRNAs or long non-coding RNAs and efficiently deliver their cargo in targeted cells [131, 262], whereas RLPs can be used for stable interfering RNA expression in cancer cells [263, 264]. Successful *in vivo* vectorization of siRNAs against the epigenetic regulator HDAC1 [265] or the viral oncogene E6 [266] was also achieved with OVs and was associated with prolonged survival in models of metastatic melanoma or cervical cancer, respectively.

To conclude, all nanovector families are investigated either in preclinical studies or clinical trials for the delivery of nucleic acids for cancer therapy (Table 3). On the one hand, gene therapy approaches are dominated by viral vectors (e.g. VLPs, OVs) (Table 2) due to their natural abilities to deliver to the nuclear compartment therapeutic transgenes that will be efficiently expressed. On the other hand, the efficient delivery of RNA molecules has been demonstrated for almost all types of nanovectors described in this review. EVs naturally transport small RNAs and present a high biocompatibility, but lipid-based nanoparticles, bacterial minicells and viruses are also adapted to such vectorization. With the expected boom of cancer gene therapies in the next few years, upcoming clinical studies will provide critical data to determine which vectors are the best compromise when considering efficient nucleic acid delivery, biocompatibility and ultimately clinical efficacy.

### Tumor microenvironment modulation & immunotherapy

In recent years, cancer treatment has rapidly evolved from directly targeting malignant cells to treating the TME as a whole [267, 268]. The stromal and immune compartments that constitute this complex environment support cancer growth, maintenance, resistance and recurrence and can be targeted for destruction or reprogramming. New technologies like single-cell profiling continuously provide a better understanding of this tumor heterogeneity and help both deciphering the intertwined mechanisms involved and developing new rationale-based therapies to target them. This is perfectly illustrated by the breakthrough of cancer immunotherapies that use either immune activating signals (e.g. cytokines, agonist antibodies) or inhibitors of immunomodulating cues (e.g. immune checkpoint inhibitors). Nevertheless, limiting off-target toxicities and moderate efficacies call for improved vectorization to further refine these approaches. Nanovectors can modulate the pharmacokinetics of immunotherapies, deliver locally combination therapies and sometimes display an intrinsic therapeutic potential [269, 270] (Table 2).

- ***Removing life support***

Cancer-associated fibroblasts (CAFs) and tumor-associated macrophages (TAMs) secrete immunomodulatory cytokines, growth factors and pro-angiogenic molecules that participate in tumor maintenance [267, 268]. A valid strategy would consist in eliminating these stromal cells, for instance by using targeted nanoparticles to specifically deliver chemotherapies and/or photosensitizers to CAFs [271–274] or bisphosphonates and other cytotoxic molecules to TAMs [269, 275]. In these approaches, nanoparticles are actively targeted to CAFs and TAMs, mostly with FAP- or αSMA-specific molecules, or with mannose moieties, respectively. OVs have also been used for anti-CAF bispecific T cell engagers (BiTEs) delivery to selectively mediate CAF death via T cell activation [276, 277]. Interestingly, the use of OVs, which infect malignant cells and replicate in the TME, allows for continuous local production of anti-CAF BiTEs by infected tumor cells. OVs can also be addressed directly to CAFs by exploiting CAF-specific promoters [278] or receptors [279] as shown with an adenovirus and measles virus, respectively.

Endothelial cells are other important actors of the TME as they ensure nutrient and oxygen supply to growing tumors. To induce tumor cell death, the tumor vasculature can thus be impaired by vectorizing anti-angiogenics, mostly VEGF inhibitors or anti-VEGF siRNAs. Those have been developed as single agents over the last two decades but showed major side effects, such as hemorrhages or thromboses [280]. Anti-angiogenics have been vectorized efficiently with nanoparticles [280], bacterial minicells [61] and OVs [281], either by active targeting to the tumor endothelium (e.g. iRGD peptide) or by relying on the EPR effect. As an example, untargeted liposomes were used to co-deliver an anti-VEGF siRNA and etoposide and caused a significant inhibition of tumor growth in an orthotopic non-small cell lung cancer model compared to the combinations of either free drugs or the separate liposomal formulations [282]. Similarly, the anti-VEGF antibody bevacizumab and erlotinib were co-vectorized in pH-sensitive lipid-polymer hybrid nanoparticles and achieved significant inhibition of non-small cell lung cancer growth in mice [283].

b. ***Reprogramming the environment***

Normalizing the TME by modifying the phenotypes and functions of its cellular components has become a therapeutic strategy to beat cancer [284]. Since reprogramming myeloid cells toward anti-tumor phenotypes can promote favorable immune responses, several strategies aim at re-educating TAMs into pro-inflammatory M1-like macrophages [285]. This can be achieved using pro-inflammatory cytokines (e.g. IL-12), miRNAs or TLR agonists which systemic delivery was shown to be highly toxic unless vectorized by nanoparticles [285–288] or VLPs [289]. Other types of immunosuppressive cell types such as myeloid-derived suppressor cells or regulatory T cells can also be targeted by engineered nanoparticles [275] and OVs [290].

OVs display intrinsic properties (e.g. induction of immunogenic tumor cell death (ICD), release of damage- and pathogen-associated molecular patterns) that make them perfectly suited for such reprogramming approaches in cancer immunotherapy. Clinical trials reported that OV-induced ICD can be sufficient to induce an abscopal anti-cancer immune response and lead to tumor eradication [94, 291, 292]. OV infection also promotes T cell infiltration in the infected tumors and could improve the efficacy of immune checkpoint inhibitors [293]. The vectorization of immunomodulating transgenes with OVs or VLPs turns cancer cells into therapeutic factories within the TME [86, 294] as shown with immune checkpoint inhibitors encoded from engineered viruses [295, 296]. This changes the pharmacokinetics of immunotherapies and enables the use of potent immune activators (e.g. trimerized CD137L, IL-12) that are toxic or even lethal when used systematically without proper vectorization. It also facilitates combinations, for example by inserting into large DNA virus genomes multiple immunotherapeutic transgenes (e.g IL-12 + anti-PD-L1) targeting different immune mechanisms for synergistic effects with no additional toxicity [296–298].

To vectorize immunotherapies targeting the TME [126], nanoparticles are generally combined with ICD inducers (e.g. hyperthermia) on the same vector in order to stimulate immune cell recruitment and activation [269, 299–301]. Contrary to transgene vectorization by OVs, nanoparticles usually transport proteins, which does not allow spatial and temporal treatment amplification. Nevertheless, inhibitors of IL-10, TGF-β, indoleamine 2,3-dioxygenase immunosuppressive molecules [273], TLR agonists [302–304] or pro-inflammatory cytokines (e.g. IL-2, IL-15, TNF-α, IFN-γ) [15, 305–308] have been successfully addressed to the TME in preclinical models using different types of nanoparticles [275, 309]. Those have also been used to vectorize anti-OX40 [310] and anti-CD137 [311] agonist antibodies or anti-PD-1 [310] and anti-PD-L1 [312] antagonist antibodies in mice to enable efficient T cell activation in the TME [270, 299]. In an elegant study, a tritherapy consisting in an immune checkpoint inhibitor (i.e. anti-PD-L1) and two T cell activators (i.e. anti-CD3 and anti-CD28) conjugated on the same nanoparticle was shown to augment the therapeutic index of the combination against murine breast and colorectal cancers [313], which illustrates the versatility of nanoparticles in this context.

Recent clinical advances in cancer immunotherapy and TME reprogramming are yet to be enhanced efficiently by appropriate vectorization approaches. Viruses display natural abilities (e.g. transgene transport and expression, intrinsic immunogenicity) for this, with OVs also exhibiting replication and oncolysis properties that can further improve their therapeutic efficacy. The development of clinical-grade viruses may be however challenging and organic nanoparticles, which are investigated in numerous preclinical studies to deliver immunomodulating proteins to tumors, offer good alternatives when considering their multiple engineering possibilities. The most efficient designs are still to be identified in clinical studies but advances in vaccination strategies using nanoparticles, for instance regarding Covid-19, may accelerate these developments. As for VLPs, EVs and bacterial minicells, their ability to vectorize biomolecules to modulate the TME has been demonstrated but clinical evidence is still missing.

## Conclusion

The last three decades have seen the discovery of a tremendous number of new anti-cancer molecules selected for their tumor-specific cytotoxicity and, more recently, for their ability to alter the TME. However, a large majority of the molecules identified on the bench fail in the clinic because of a poor efficacy/safety ratio after systemic administration. Despite personalized combinations to strike tumors on different fronts, resistance and toxicities are still major issues that limit many therapeutic applications. The advent of nanotechnologies opened an entirely novel area of research around the nanovectorization of anti-tumor therapeutics to both increase treatment efficacy and reduce associated toxicities by improving dramatically the specificity of tumor targeting. Chemically engineered nanoparticles – highly adaptable and for some relatively easy to manufacture – were the first to enter the clinic but with the current trend to improve the biocompatibility and to exploit precise biological mechanisms, bio-inspired nanovectors (e.g. VLPs, bacterial minicells, EVs, OVs) are now rapidly gaining interest. These different families of nanovectors allow the vectorization of almost all anti-cancer therapeutics, including chemical drugs, radio-elements, nucleic acids, toxins and immunotherapies (Table 2). To this day, chemotherapies, radioelements and molecules that sensitize tumors to radiotherapies have been more efficiently vectorized with synthetic nanoparticles but promising results have also been obtained with bacterial minicells and VLPs. By their very nature, viral vectors are the most suitable for gene therapy and nucleic acid vectorization, yet lipid-based nanoparticles have been extensively studied for these applications and may be more adapted – along with EVs or even bacterial minicells – to the delivery of small RNAs. Finally, nanoparticles can efficiently vectorize immunomodulatory proteins but OVs are becoming a new standard thanks to their intrinsic immunogenic properties and their ability to sustain local expression of immunomodulatory transgenes.

The field of nanovectorization is overly active and has already provided important advances for cancer therapy, with clinical approvals for several simple nanoformulations (Table 3). Current developments however focus on more complex structures including biological or bio-inspired objects. This opens opportunities for the advancement of personalized medicine by adapting rationally the nanovectors to specific biological contexts and clinical situations, but this also comes with several hurdles on the way to clinical applications. Indeed, the increasing complexity of synthetic nanoparticles, in particular for combination therapies, will necessitate radical optimization of production methods. For the bio-inspired nanovectors, the issues associated with the cost and the technical difficulties of large-scale productions still hinder their wider development. Moreover, the nanovectorization of anticancer therapeutics also lacks solid pharmacological and toxicological studies; improvements and solutions may come from advances in parallel fields such as recombinant protein production, conventional gene therapy or regenerative medicine. These problems highlight the importance of integrating the issue of therapeutic delivery in the process of drug development and call for a closer relationship with the field of drug discovery. As such, acknowledging the diversity of available delivery systems may act as a lever in drug discovery and reveal numerous therapeutic molecules that would have been rejected because of alleged unfavorable properties (e.g. poor solubility, high toxicity), thereby expanding the therapeutic arsenal against cancer.

## Data Availability

Not applicable.

## Abbreviations

AAV: adeno-associated virus
AuNP: gold nanoparticle
CAF: cancer-associated fibroblast
ECM: extracellular matrix
EGFR: epidermal growth factor receptor
REPR: enhanced permeability and retention
EV: extracellular vesicle
GDEPT: gene-directed enzyme/prodrug therapy
MDR: multidrug resistance
NIS: sodium/iodide symporter
NO: nitric oxide
OV: oncolytic virus
PAA: polyacrylic acid
PDT: photodynamic therapy
PEG: polyethylene glycol
pHLIP: pH (low) insertion peptide
RLP: retrovirus-like particle
RNAi: RNA interference
ROS: reactive oxygen species
SPION: super paramagnetic iron nanoparticle
TAM: tumor-associated macrophage
TME: tumor microenvironment
VLP: virus-like particle
